# Editorial: Dopamine Neuron Diversity in Circuits and Diseases

**DOI:** 10.3389/fncir.2022.856716

**Published:** 2022-03-02

**Authors:** Jean-Francois Poulin, Talia Newcombe Lerner, Mark W. Howe

**Affiliations:** ^1^Montreal Neurological Institute, McGill University, Montreal, QC, Canada; ^2^Department of Physiology, Feinberg School of Medicine, Northwestern University, Chicago, IL, United States; ^3^Department of Psychological and Brain Sciences, Boston University, Boston, MA, United States

**Keywords:** dopamine, circuit, mesocortical dopamine system, addiction, Parkinson's Disease, Autism Spectrum Disorder (ASD)

In 1957–1958, Arvid Carlsson demonstrated that dopamine (DA) acts as a neurotransmitter in the brain and pinpointed its crucial role in Parkinson's Disease (PD; Carlsson et al., 1957, 1958). This ground-breaking discovery, which won the Nobel Prize in 2000, led to the adoption of the DA precursor L-DOPA as a life-changing treatment for PD. Further technological developments such as the Falck-Hillarp fluorescence histochemical method (Dahlstrom and Fuxe, 1964), which allowed the visualization of monoamines in histological sections, and the discovery of the neurotoxin 6-hydroxydopamine (Uretsky and Iversen, 1970), which selectively targets DAergic and noradrenergic neurons, enabled identification of the three major ascending DAergic pathways of the mammalian brain: the nigrostriatal, mesocortical, and mesolimbic pathways (Moore and Bloom, 1978). In the last several decades, it has become increasingly apparent that diverse DA neuron populations exist within and across these three pathways which differ in their molecular signatures, developmental origins, physiological characteristics, anatomical connections, and functional roles during behavior. New sequencing approaches are revealing significant molecular diversity in DA neurons that has led to classifications of new subtypes with unique physiology and connectivity. Advances in *in-vivo* monitoring approaches are demonstrating that different DA neuron populations and their projections in the striatum transmit signals related to diverse aspects of rewarding and aversive outcomes, sensory stimuli, and behavior.

How DA neuron diversity impacts downstream striatal or cortical circuits, and how dysfunction in particular DA neurons subpopulations generates the symptoms of neurological and psychiatric disorders as disparate as PD, autism spectrum disorder, and addiction are currently Research Topics of intense investigation. Here, we present a Research Topic that contains a collection of both original and review articles illustrating the diversity of the midbrain DAergic system. This Research Topic of nine articles highlights key topics in the neuroanatomical, molecular, developmental, and functional determinants of DA diversity.

Fundamental differences in the physiological and wiring properties of DA neurons within individual neuroanatomical structures such as the substantia nigra pars compacta (SNc) and ventral tegmental area (VTA) are increasingly appreciated. The work of Montero et al. demonstrates such differences by characterizing the dendritic architecture and firing properties of DA neurons located within these structures. Their results suggest dendritic morphology shapes both the physiological properties and connectivity of DA neurons within a single neuroanatomical structure. Similarly, the article from Derdeyn et al. illustrates the diversity of synaptic input to VTA neurons and provides an in-depth analysis of rabies-based circuit mapping studies of the VTA. In addition, Eskenazi et al. provide a systematic review of DA neurons expressing the glutamate vesicular transporter Vglut2, which includes the synaptic properties and connectivity of these neurons.

Abnormal development of the DAergic system has a broad impact on behavior and vulnerability to neuropsychiatric diseases. Two articles provide an in-depth review of the early development and the maturation of the mesocortical pathway, contributed by Islam et al. and Reynolds and Flores, respectively. These articles summarize the current state of the field but also highlight windows of vulnerability to psychiatric and neurological conditions.

The DAergic system is particularly vulnerable and can be hacked by drugs of abuse to produce self-destructive behavioral patterns. The role of diverse DA pathways in substance abuse and addiction is expertly reviewed by Poisson et al., not an easy task due to the long history of this literature. In addition, the implications of DA neuron diversity in response to the drug of abuse amphetamine was investigated by Serra et al. focussing on a medial VTA subpopulation that expresses the gene *TrpV1*. Among other findings, Serra et al. demonstrate that removing the ability of TrpV1+ neurons to package and release DA leads to sensitization of the response to amphetamine.

Finally, the role of DA neuron diversity in neurological diseases is the Research Topic of two excellent articles. Kosillo and Bateup provide a nice overview of the DAergic dysfunction observed in genetic mouse models of Autism Spectrum Disorders (ASD). Although it is increasingly clear that DA plays a significant role in symptoms of ASD, the authors conclude that more investigations are needed if a complete picture of the role of diverse DA populations is to emerge. On the other hand, the picture of DA diversity in PD is clearer as a subtype of DA neuron, defined by the expression of *Aldh1a1* and located in the SNc, appears to be selectively vulnerable in PD. Carmichael et al. review what is currently known about this DA neuron population and its role in motor control and PD.

Overall, tremendous progress has been made since the pioneering work of Carlsson. Discoveries regarding the rich diversity within the midbrain DA system are both fascinating and necessary for the further development of treatments for an array of neurological and psychiatric disorders. We are indebted to the authors for their contributions to this Research Topic and hope you'll enjoy reading these articles as much as we did.

## Author Contributions

All authors listed have made a substantial, direct, and intellectual contribution to the work and approved it for publication.

## Conflict of Interest

The authors declare that the research was conducted in the absence of any commercial or financial relationships that could be construed as a potential conflict of interest.

## Publisher's Note

All claims expressed in this article are solely those of the authors and do not necessarily represent those of their affiliated organizations, or those of the publisher, the editors and the reviewers. Any product that may be evaluated in this article, or claim that may be made by its manufacturer, is not guaranteed or endorsed by the publisher.
